# Timing of delivery in a high-risk obstetric population: a clinical prediction model

**DOI:** 10.1186/s12884-017-1390-9

**Published:** 2017-06-29

**Authors:** Dane A. De Silva, Sarka Lisonkova, Peter von Dadelszen, Anne R. Synnes, Canadian Perinatal Network (CPN) Collaborative Group, Laura A. Magee

**Affiliations:** 1 Department of Obstetrics & Gynaecology, University of British Columbia, C420-4500 Oak Street, Vancouver, BC V6H 3N1 Canada; 2 School of Population and Public Health, University of British Columbia, 2206 E. Mall, Vancouver, BC V6T 1Z9 Canada; 3 St. George’s University Hospitals NHS Foundation Trust, Blackshaw Road, Tooting, London, SW17 0QT UK; 4 Molecular & Clinical Sciences Research Institute, St. George’s University of London, Rm J0.27, Jenner Wing, Cranmer Terrace, London, SW17 0RE UK; 5 Division of Neonatology, Department of Paediatrics, University of British Columbia, 1R14-4500 Oak Street, Vancouver, BC V6H 3N1 Canada

**Keywords:** Preterm birth, Prediction model, Antenatal corticosteroids

## Abstract

**Background:**

The efficacy of antenatal corticosteroid treatment for women with threatened preterm birth depends on timely administration within 7 days before delivery. We modelled the probability of delivery within 7 days of admission to hospital among women presenting with threatened preterm birth, using routinely collected clinical characteristics.

**Methods:**

Data from the Canadian Perinatal Network (CPN) were used, 2005–11, including women admitted to hospital with preterm labour, preterm pre-labour rupture of membranes, short cervix without contractions, or dilated cervix or prolapsed membranes without contractions at preterm gestation. Women with fetal anomaly, intrauterine fetal demise, twin-to-twin transfusion syndrome, and quadruplets were excluded. Logistic regression was undertaken to create a predictive model that was assessed for its calibration capacity, stratification ability, and classification accuracy (ROC curve).

**Results:**

We included 3012 women admitted at 24–28 weeks gestation, or readmitted at up to 34 weeks gestation, to 16 tertiary-care CPN hospitals. Of these, 1473 (48.9%) delivered within 7 days of admission. Significant predictors of early delivery included maternal age, parity, gestational age at admission, smoking, preterm labour, prolapsed membranes, preterm pre-labour rupture of membranes, and antepartum haemorrhage. The area under the ROC curve was 0.724 (95% CI 0.706–0.742).

**Conclusion:**

We propose a useful tool to improve prediction of delivery within 7 days after admission among women with threatened preterm birth. This information is important for optimal corticosteroid treatment.

**Electronic supplementary material:**

The online version of this article (doi:10.1186/s12884-017-1390-9) contains supplementary material, which is available to authorized users.

## Background

Preterm birth is the leading cause of perinatal mortality and morbidity in Canada and worldwide [1, 2]. One the most effective means to reduce neonatal mortality and morbidity in preterm infants is antenatal administration of corticosteroids [3, 4]. The proven benefits of antenatal corticosteroids for fetal lung maturation and prevention of serious neonatal morbidity among women with preterm delivery have resulted in the inclusion of corticosteroid treatment as standard obstetric care in industrialised countries [3, 5–7]. Treatment guidelines recommend administering corticosteroids to women who are at risk of delivering within the next 7 days when they present between 24^+0^ and either 33^+6^ weeks gestation in Canada [5] and the United States [6], or up to 35^+6^ weeks gestation in the United Kingdom [7].

One of the major barriers to appropriate use of antenatal corticosteroids is that timing of delivery is often unknown; approximately half of women who are admitted to hospital with threatened preterm birth remain undelivered after 7 days [8, 9]. This makes it difficult to maximise ‘optimal’ use of corticosteroids (i.e., administration to women who go on to deliver within the next 7 days and not administering them to women who do not deliver within the next 7 days). A recent study from Nova Scotia, Canada, showed that between 1988 and 2012, the proportion of women with suboptimal antenatal corticosteroid treatment (i.e., more than 7 days prior to delivery) increased approximately 5 times (from 7% to 34%) whereas optimal treatment doubled (from 10% to 23%) [10].

Until recently, administration of a single course of steroids at <34 weeks was considered both effective and safe. Increasingly, however, concerns have emerged about adverse effects following multiple courses and side effects following a single course administered in the community in under-resourced settings [11–17]. Multiple antenatal corticosteroid exposure at preterm gestation has been found to affect fetal growth and CNS development [11–14], and to be associated with neurosensory dysfunction in particular among infants born at term [15, 16]. In a recent cluster randomised controlled trial in low- and middle-income countries (LMICs), community administration of antenatal corticosteroids for threatened preterm birth before 36 weeks was associated with no benefit of antenatal corticosteroids among babies born preterm, and with a 12% increase in neonatal mortality and a 45% increase in suspected maternal infection [17].

Actual corticosteroid treatment rates vary widely, depending in part on maternal and obstetrical characteristics [4] and the difficulty in predicting preterm birth [18, 19]. Most literature focuses on predictors as individual tests [20, 21] or clinical biomarkers; [20, 22] however, they have not been found to be as useful in predicting preterm birth among asymptomatic or nulliparous women, even when combined [21–24]. There is a paucity of literature on prediction models that combine multiple determinants of preterm birth, particularly within 7 days of admission. Moreover, there is a lack of models that use characteristics that are available upon admission. Thus, based on clinical characteristics collected in routine clinical practice, we aimed to identify women who were likely to deliver within 7 days after admission to hospital due to a high-risk of preterm delivery.

## Methods

We carried out a retrospective cohort study of women admitted to any of the 16 perinatal centers participating in the Canadian Perinatal Network (CPN) (Additional file 1). The CPN study collected demographic, clinical, and birth information on women who were both at high-risk of very preterm birth between 22^+0^ and 28^+6^ weeks gestation and admitted (or re-admitted) to the participating CPN tertiary hospitals for at least 24 h from August 1, 2005 to March 31, 2011. These women were followed-up until delivery.

The CPN database collected information on maternal demographic and behavioural characteristics (e.g., maternal age, marital status, smoking history, parity), past medical and obstetric history (e.g., number of prior abortions and preterm births), characteristics of current pregnancy (e.g., fertility treatments, use of drugs or alcohol in pregnancy, reason for admission to hospital), maternal and fetal surveillance and treatments (e.g., expectant care, use of antibiotics, or partial or completed courses and subsequent courses of corticosteroids), and pregnancy outcomes. CPN details including data definitions have been published previously [25]. Ethics approval was obtained centrally as a quality assurance project by the Research Ethics Boards at the University of British Columbia (H05–70359) and at each study site. As such, written consent was not required, and any collected information was anonymised and de-identified prior to analysis.

In the current study, we included women in the CPN database who presented between 24^+0^ and 28^+6^ weeks gestation with one or more of the following conditions at enrollment: preterm labour, preterm pre-labour rupture of membranes (PPROM), short cervix without uterine contractions, dilated cervix or prolapsed membranes without uterine contractions on admission (see Additional file 2 for definitions). We also included women who were admitted for these conditions occurring de novo at up to 34^+6^ weeks after previous admission to the CPN network for other reasons, as these women would also be eligible to receive corticosteroids. We excluded women whose pregnancies were complicated by intrauterine fetal demise at the time of admission, twin-to-twin transfusion syndrome, those with quadruplets, and known fetal anomaly due to differences in their clinical management [26].

To create a generalisable model, it is recommended that highly subjective factors or those dependent on the health care system, local practice, or individual practitioners should not be considered as predictors [27, 28]. Therefore, we did not include the following variables as candidate predictors in our study: transfer to another facility, use of cervical cerclage, tocolytic and antibiotic treatment, and the fetal fibronectin test administered before admission.

Unadjusted associations between candidate predictors and delivery within 7 days were tested using chi-square and Fisher’s exact test (as appropriate for categorical variables), or the Student’s t-test or Wilcoxon non-parametric test (as appropriate for continuous variables). For the initial multivariable logistic regression model, we selected candidate predictors that were either associated with delivery within 7 days at *p* < 0.10 [29], or for face validity, were known to be clinically relevant to preterm birth (including alcohol use during pregnancy, and previous preterm birth) (Table 1) [30–32]. Potential interactions between candidate predictors were examined. In the multivariable model, the association between delivery within 7 days and candidate predictors that were continuous (i.e., maternal age and gestational age) was examined using clinically meaningful categories, product terms, and splines where necessary. Missing values for all variables in the model were imputed using the MICE method of multiple imputation [29, 33]. This method assumes data are missing at random; to test this assumption, the cases with complete data were compared to cases with missing variables. Imputation models were built including all possible predictors of the variable to be imputed and the outcome variable. This was done five times to generate five completed datasets with plausible values for the missing variable.

**Table 1:** Demographic and clinical characteristics of women admitted to a tertiary hospital at 24–34 weeks gestation

	Delivery within 7 days (*N* = 1473)	Delivery in >7 days (*N* = 1539)	*p* value﻿﻿^﻿a^
Baseline Demographic and Medical/Surgical History			
Maternal age on admission (years)^a^			
< 20	74 (5.0%)	45 (2.9%)	
20–24	219 (14.9%)	213 (13.8%)	
25–29	403 (27.4%)	404 (26.3%)	
30–34	466 (31.6%)	509 (33.1%)	**0.002**
35–39	260 (17.7%)	275 (17.9%)	
40–44	43 (2.9%)	79 (5.1%)	
≥ 45	8 (0.5%)	14 (0.9%)	
Nulliparous^a^	811 (55.1%)	699 (45.4%)	**<0.001**
Prior miscarriage^a^	432 (29.3%)	514 (33.4%)	**0.018**
Prior therapeutic abortion	227 (15.4%)	231 (15.0%)	0.798
Prior birth at <37 weeks	213 (14.5%)	321 (20.9%)	**0.013**
Prior birth at 34–36 weeks^a^	82 (5.6%)	131 (8.5%)	**0.090**
Prior birth at <34 weeks^a^	161 (10.9%)	190 (12.3%)	0.476
Pre-existing medical conditions			
Pre-existing hypertension^a^	26 (1.8%)	48 (3.1%)	**0.023**
Pre-existing diabetes mellitus	20 (1.4%)	24 (1.6%)	0.757
Uterine structural abnormalities^a,c^	73 (5.0%)	94 (6.1%)	0.193
Renal disease/urology^a^	7 (0.5%)	20 (1.3%)	**0.027**
Rheumatic disease	14 (1.0%)	8 (0.5%)	0.241
Cardiac disease	10 (0.7%)	14 (0.9%)	0.612
Cervical procedures	17 (1.2%)	21 (1.4%)	0.723
Other^b^	202 (13.7%)	261 (17.0%)	0.016
Smoking after pregnancy diagnosed^a^	321 (21.8%)	265 (17.2%)	**0.002**
Missing	18 (1.2%)	5 (0.3%)	
Alcohol use during pregnancy (socially or at least twice weekly)^a^	43 (2.9%)	42 (2.7%)	0.838
Missing	19 (1.3%)	11 (0.7%)	
Illicit drug use after pregnancy diagnosed^a^	69 (4.7%)	52 (3.4%)	0.083
Missing	19 (1.3%)	7 (0.5%)	
Current pregnancy			
GA on admission^a^ (weeks)			
24 + ^0^–25 + ^6^	520 (35.3%)	602 (39.1%)	
26 + ^0^–27 + ^6^	593 (40.3%)	616 (40.0%)	
28 + ^0^–29 + ^6^	329 (22.3%)	311 (20.2%)	**<0.001**
30 + ^0^–31 + ^6^	10 (0.7%)	7 (0.5%)	
32 + ^0^–34 + ^6^	21 (1.4%)	3 (0.2%)	
Multiple pregnancy^a^	256 (17.4%)	380 (24.7%)	**<0.001**
Twins	237 (16.1%)	343 (22.3%)	
Triplets	19 (1.3%)	37 (2.4%)	
Reasons for admission			
Preterm labour^a^	776 (52.7%)	489 (31.8%)	**<0.001**
Preterm pre-labour rupture of membranes^a^	597 (40.5%)	483 (31.4%)	**<0.001**
Short cervix without contractions^a^	125 (8.5%)	548 (35.6%)	**<0.001**
Dilated cervix or prolapsed membranes without contractions^a^	241 (16.4%)	154 (10.0%)	**<0.001**
Other associated complications/conditions at admission			
Antepartum haemorrhage^a^	219 (14.9%)	123 (8.0%)	**<0.001**
Gestational hypertension (any)	9 (0.6%)	4 (0.3%)	0.170
Gestational hypertension with proteinuria	7 (0.5%)	3 (0.2%)	0.217
Intrauterine fetal growth restriction	18 (1.2%)	25 (1.6%)	0.604

^a^Factors considered in the full model with a *p *value <0.10 (as ﻿highlighted in bold)

^b^Other conditions include intra-abdominal infection (*N* = 92), asthma (*N* = 162), neurologic disease (*N* = 47), STDs (*N* = 82), gastrointestinal disease (*N* = 23), liver disease (*N* = 36), pre-existing thrombophilia (*N* = 22), pre-existing thromboembolism (*N* = 17), psychiatric disorders (*N* = 132), or other underlying medical conditions (*N* = 27)

^c^Includes leiomyomas (*N* = 84), bicornuate uterus (*N* = 54), unicornuate uterus (*N* = 5), didelphic uterus (*N* = 13), and other (*N* = 16)

Backward selection was used to remove candidate predictors that were not significantly associated with birth within 7 days (Wald statistic *p* > 0.05) to obtain the final model [34].

The diagnostic performance of the final model was assessed in terms of calibration capacity, stratification capacity, and classification accuracy [34]. Our goal was to develop a pragmatic model with high sensitivity, low false negative rate (of <5% based on the precedent set by prenatal diagnostic screening) [35–37], and also high negative predictive value (NPV), to best identify women for whom an immediate administration of steroids may be suboptimal. Calibration capacity was assessed by a calibration curve that indicated whether the proportion of women who delivered within 7 days in each group of deciles of modeled probability corresponded to the predicted probability. Risk stratification capacity was assessed by a classification table that showed whether the model had the capacity to distinguish between high- and low-risk groups, and whether these categories were clinically meaningful. Finally, classification accuracy was assessed by the extent to which the women who delivered within 7 days had an increased predicted probability of doing so, and whether women who delivered beyond 7 days had a low predicted probability of delivery within 7 days (i.e., sensitivity and specificity, receiver operating characteristic [ROC] curve). Internal validation of the model was performed using a bootstrap method on the model development set with 200 iterations to generate bias-corrected ROC and calibration curves.

Sensitivity analyses included assessment of other pre-existing medical and/or surgical conditions that may be associated with preterm birth, and the impact of missing values on the predictive performance of the model by excluding these cases. To ensure that the model performed well for subgroups of women that may be under-represented in our population, we tested the final model’s accuracy separately for women with singleton vs. multiple gestation, and for women admitted at 24–31 weeks vs. 32–34 weeks gestation. All statistical analyses were performed using R 3.1.1 (http://www.r-project.org).

## Results

There were 3012 women with a primary admission at 24^+0^ to 28^+6^ weeks gestation or with a subsequent admission up to 34^+6^ weeks gestation (after a previous admission for other indications) who were eligible for the study. Of these, 1473 (48.9%) women delivered within 7 days of admission, while 1539 (51.1%) women delivered after 7 days. Approximately one-third of women (31.1%) delivered within 48 h (see Additional file 3), and 14.2% of women delivered at term gestation. Among women who delivered within 7 days of admission (*N* = 1473), 846 (57.4%) received steroids on admission to hospital. Among those who delivered more than 7 days after admission (*N* = 1539), 941 (61.1%) received steroids on admission to hospital and 598 (38.9%) did not receive steroids on admission to hospital. Thus, 1444 (47.9%) women received optimal therapy on admission with respect to steroids (by either receiving it and delivering within 7 days [*N* = 846], or not receiving it and not delivering within the next 7 days [*N* = 598]) and 1568 (52.1%) women received suboptimal therapy on admission (by not receiving steroids and delivering within 7 days [*N* = 627], or receiving steroids and delivering more than 7 days later [*N* = 941]). In routine practice, however, it is recognized that a waiting period may be used after admission because ongoing assessment allows for re-evaluation of the need for antenatal corticosteroids. In our study, 74 women did receive antenatal corticosteroids after admission (day 1–7) and delivered within 7 days, while 138 women received antenatal corticosteroids after admission and delivered more than 7 days after the steroid administration (decreasing the overall optimal use to 45.6% and increasing overall suboptimal use to 54.4% in this cohort).

Demographic characteristics and obstetric history, current pregnancy characteristics up to the time of admission, and maternal interventions during hospitalization among women who delivered within 7 days from admission vs. those who delivered later are described in Table 1. Women who delivered within 7 days after admission were more likely to be younger, primiparous, have a history of spontaneous abortion and preterm birth, have a singleton pregnancy, smoke during pregnancy, and be admitted at higher gestational age. These women were also more likely to be admitted for preterm labour, PPROM, and dilated cervix or prolapsed membranes, and less likely to be admitted with short cervix without contractions. They were more likely to have associated antepartum haemorrhage.

As expected, adverse perinatal and maternal health outcomes occurred more frequently among women who delivered within 7 days, compared with those who delivered later (see Additional file 4). Only 200 (6.6%) women had fibronectin testing data available, and 252 (8.4% overall, or 37.4% among women with a short cervix) had a rescue or elective cerclage for short cervix.

**Fig. 1:**
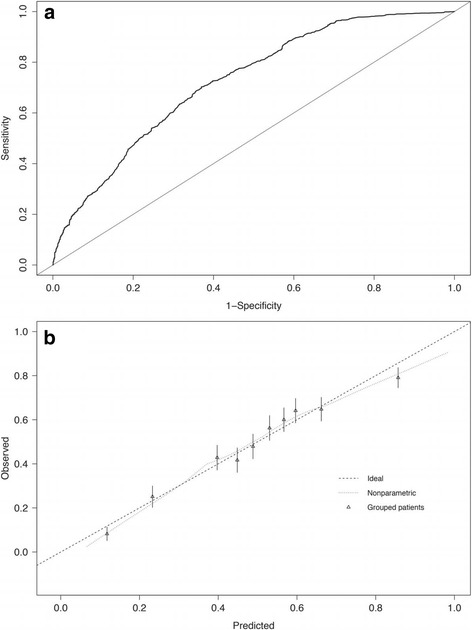
Graphical presentation of prognostic performance of the final model to predict delivery within 7 days of admission among a high-risk cohort. **a** Area under the ROC curve. **b** Calibration curve

There were no significant interactions identified between candidate predictors. The final model included eight variables whose associations with delivery within 7 days were significant. The crude and adjusted odds ratios and 95% confidence intervals are presented in Table 2. The predictive model equation was as follows: Risk score =12.13 – [0.41 × Maternal age ≥ 40years] – [1.08 × GA] + [0.02 × (GA)^2^] – [0.54 × Parity] + [0.32 × Smoking] + [2.00 × Preterm labour] + [1.72 × PPROM] + [1.85 × Prolapsed membranes] + [0.67 × Antepartum haemorrhage]; the probability of outcome (delivery <7 days) = 1/(1 + e^-risk score^). Based on this model, for example, a woman who is 35 years old, nulliparous, non-smoking, and presents at 28 weeks’ gestation with PPROM at admission would have, on average, a 34% probability of delivery within 7 days, whereas the same woman presenting with PPROM and preterm labour would have a probability of 79%. Figure 1a displays a ROC curve for the final model, with AUC = 0.724 (95% CI: 0.706–0.742).

**Table 2:** Risk factors included in the final model predicting delivery within 7 days of admission

Risk factor	OR [95% CI]	Adjusted OR^a^ [95% CI]
Maternal age (yr)		
< 40	Reference	Reference
≥ 40	0.55 [0.39–0.78]	0.66 [0.45–0.97]
Parity		
Nulliparous	Reference	Reference
Parity ≥1	0.68 [0.59–0.78]	0.58 [0.50–0.68]
Smoking during pregnancy^b^	1.35 [1.13–1.62]	1.37 [1.12–1.67]
Gestational age (GA) on admission^c^	1.08 [1.03–1.13]	^c^
Maternal conditions		
Preterm labour	2.38 [2.05–2.76]	7.37 [5.85–9.29]
PPROM	1.52 [1.30–1.76]	5.59 [4.42–7.07]
Prolapsed membranes	1.75 [1.41–2.18]	6.36 [4.77–8.48]
Associated antepartum haemorrhage	2.06 [1.63–2.60]	1.96 [1.53–2.52]

*OR* odds ratio, *PPROM* preterm pre-labour rupture of membranes

^a^adjusted for all other factors presented in the table

^b^missing values were imputed

^c^Gestational age was modelled using higher order polynomials (see the equation below)

Equation:

Risk score =12.13 – [0.41 × Maternal age ≥ 40years] – [1.08 × GA] + [0.02 × (GA)^2^] – [0.54 × Parity] + [0.32 × Smoking] + [2.00 × Preterm labour] + [1.72 × PPROM] + [1.85 × Prolapsed membranes] + [0.67 × Antepartum haemorrhage]

the probability of outcome (delivery <7 days) = 1/(1 + e^-risk score^)

The calibration curve (Fig. 1b) showed good calibration capacity of the final model, as the proportion of women who delivered within 7 days in each risk category corresponded to the predicted risk. Predicted probabilities that were very low (<20%) and high (>80%) were slightly overestimated.

The final model had a relatively good calibration and stratification ability to distinguish between women with high vs. low risk of delivering within 7 days (Table 3). Among women with 0–15% predicted probability of delivery within 7 days, 6% delivered within 7 days, while among those with ≥65% predicted probability, 75.4% delivered within 7 days. Among women who delivered within 7 days, only 1% had a low probability of such delivery, while approximately 25% had a high probability.

**Table 3:** Stratification ability of the predictive model to identify women with and without the outcome (delivery within 7 days)

	Delivery within 7 days			Delivery after 7 days			Total
Predicted probability	*N*	Calibration (% in probability category)	Stratification (% of all women with outcome)	*N*	Calibration (% in probability category)	Stratification (% of all women without outcome)	
0–14.9%	14	6.0	1.0	219	94.0	14.2	233
15.0–19.9%	34	15.1	2.3	191	84.9	12.4	225
20.0–49.9%	392	41.2	26.6	560	58.8	36.4	952
50.0–64.9%	668	59.7	45.3	450	40.3	29.2	1118
≥65.0%	365	75.4	24.8	119	24.6	7.7	484
Total	1473		100	1539		100	3012

Different thresholds of probability were considered to identify women unlikely to deliver within 7 days. Table 4 shows a good stratification ability of the model with meaningful probability categories. We used probability cut-offs as follows: ≤15% as our lowest threshold in order to have a sufficient number of women included in this category, <27% to minimise the false negative rate to <5% (based on similar prenatal diagnostic testing criteria) [35, 38], <50% to be better than the flip of a coin, and ≥65% a threshold with a sufficient number of women in this ‘high probability of delivery’ category. If the optimal cut-off point were to maximise negative predictive probability (NPV) with a chosen acceptable false negative rate of <5%, the optimal threshold of predicted probability would be ≤27% (sensitivity 96.2% with the lower 95% CI limit of 95.1%; NPV of 89.0%, 95% CI 86.0–91.5). At this threshold, corticosteroid treatment would be given to 2502 (83.1%) women and withheld from 510 (16.9%), with treatment that would be optimal in 1871 (62.1%) women and suboptimal in 1141 (37.9%), a significant difference compared with values in our cohort of 47.9% and 52.1%, respectively (*p*-value <0.001). The corresponding negative likelihood ratio of 0.13 (95% CI 0.10–0.17) indicates a highly informative test in terms of ruling out delivery within 7 days. However, in practice, the predicted probability of delivery within 7 days would best be used as a continuous value to customise management.

**Table 4:** Prognostic accuracy of delivery within 7 days at various cut-off points of predicted probability

Predicted probability (%)	<15.0^a^	<27.0	<50.0	<65.0
Number of women	233	510	1410	2528
N women delivered at ≤7 days (*N* = 1473 overall)	14	56	440	1108
N women delivered at >7 days (*N* = 1539 overall)	219	454	970	1420
Sensitivity	99.1% (98.4–99.4)	96.2% (95.1–97.1)	70.1% (67.7–72.4)	24.8% (22.6–27.1)
Specificity	14.2% (12.6–16.1)	29.5% (27.3–31.8)	63.0% (60.0–65.4)	92.3% (90.8–93.5)
False positive rate	85.8% (83.9–87.4)	70.5% (68.2–72.7)	37.0% (34.6–40.0)	7.7% (6.4–9.2)
False negative rate	0.9% (0.6–1.6)	3.8% (2.9–4.9%)	29.9% (27.6–32.3)	75.2% (72.9–77.4)
Positive predictive value	52.5% (50.6–54.4)	56.6% (54.7–58.6)	64.5% (62.1–66.8)	75.4% (71.4–79.0)
Negative predictive value	94.0% (90.2–96.4)	89.0% (86.0–91.5)	68.9% (66.3–71.2)	56.2% (54.2–58.1)
Positive likelihood ratio	1.16 (1.13–1.18)	1.37 (1.32–1.41)	1.90 (1.76–2.04)	3.93 (3.15–4.91)
Negative likelihood ratio	0.07 (0.04–0.11)	0.13 (0.10–0.17)	0.47 (0.43–0.52)	0.82 (0.79–0.84)

^a^e.g., if women with predicted probability <15% were considered at low-risk of delivery within 7 days, the sensitivity of the prognostic tool would be 99.1% while the specificity would be 14.2

Internal validation of the final model using a bootstrap method with 200 repetitions yielded an AUC optimism of 0.004, with bias-corrected AUC = 0.720 (95% CI 0.702–0.738). The bias-corrected calibration curve did not exhibit any appreciable changes compared with the initial curve (see Additional file 5).

Sensitivity analyses were performed including pre-existing medical and/or surgical conditions (such as renal disease, rheumatic disease, cervical procedures, cardiac disease) in the full model but were not found to be significant predictors. Sensitivity analysis was also performed excluding women with missing values. Of the variables in the final model, only smoking during pregnancy had missing values (*N* = 23); the results remained unchanged after exclusion of those cases (see Additional file 6).

We performed additional analyses to examine the accuracy of the final model among subgroups of women that may be under-represented in our population. We applied the final model to women with singleton vs. multiple pregnancy (see Additional file 7); the predictive accuracy for each subgroup was similar to the overall model performance (AUC 0.71 [0.69, 0.73] and AUC 0.77 [0.73, 0.81], respectively). Similarly, we applied the model to women admitted at 24–31 weeks vs. 32–34 weeks gestation (see Additional file 8). The predictive accuracy was similar for women admitted at 24–31 weeks (AUC 0.72 [0.71, 0.74]), however, we could not test all model parameters for the subgroup of women admitted later due to low sample size.

## Discussion

### Main findings

Our large retrospective cohort study included women admitted to a tertiary hospital for conditions that put them at high risk for preterm birth, primarily at 24^+0^ and 29^+6^ weeks gestation. Approximately 49% delivered within 7 days after admission, while 51% delivered after 7 days (14% delivered at term). We constructed a parsimonious predictive model to identify women who are at risk of delivery within the next 7 days in order to optimise administration of antenatal corticosteroids. The model had a fair predictive accuracy (AUC of 0.73), good calibration capacity and stratification ability, and it was internally validated using a bootstrap method. Although the predictive accuracy of our model was recognised to be fair, it is based on information collected in routine clinical care and it would significantly improve optimal steroid treatment on admission, (from 47.9% to 62.1% in our cohort) by decreasing the number of women who do not but should have received steroids (false negative rate). The proportion of women with optimal treatment could be maximised further with a different probability cut-off, recognising that the ideal trade-off between sensitivity and specificity will depend on the clinician’s and patient’s view of potential benefits and harms associated with either providing or postponing treatment.

### Interpretation

Optimising antenatal corticosteroid use requires accurate identification of women who will deliver within 7 days and benefit from this treatment. However, preterm birth is notoriously difficult to predict [19]. Meta-analyses of individual prognostic factors for preterm delivery showed that fetal fibronectin, absence of fetal breathing movements, and cervical length have potential for diagnostic use [20]. However, recent studies have shown that fetal fibronectin testing does not have an optimal clinical utility, as it does not prevent preterm birth nor prevent adverse perinatal outcomes among women with threatened pretem labour, and is further associated with increased cost [21, 23, 24]. Only fetal breathing movements as a prognostic test consistently yielded a likelihood ratio of a highly informative test (LR+ >10). However, these meta-analyses included very heterogeneous studies examining spontaneous preterm delivery within 24 h, 7, and 10 days among women with preterm labour [18, 20]. In contrast to our study, these predictors were examined as individual tests, and not in combination with other determinants of early preterm delivery, and as such could not be used to estimate the full range of probability of delivery. Only a few studies focused directly on predictive modeling of early delivery among selected groups of women, including those with multiple pregnancies [39] or those transferred from one hospital to a higher-level hospital [26]. The latter study identified predictors of delivery within 48 h of transfer, of which seven were similar to our result, with the addition of sonographic length of uterine cervix (a shorter length indicated a higher probability of delivery). Other studies reporting the association between preterm birth and preterm pre-labour rupture of membranes, prolapsed membranes, vaginal bleeding, cervical dilatation, preterm labour, older maternal age (>40 years), higher parity, or smoking are consistent with our results [20, 32]. However, these studies did not focus on predictive probability of preterm birth for individual women. Inclusion of these known key risk factors as predictors in our model thus increases its face validity.

### Strengths and limitations

A strength of this study is that it includes a large population dataset with detailed clinical information collected at the time of admission to the hospital with threatened preterm birth, the point in time at which clinicians must decide whether to give antenatal corticosteroids so that they will have the maximal effect by 48 h before delivery. However, there are limitations to our study. First, the time of admission was not recorded in the CPN database, and thus our outcome potentially included women delivering within 7 days and 23 h after admission. Second, the predictive model is based solely on patient and pregnancy characteristics at the time of admission and the reason(s) for admission. The predictive probability of delivery within 7 days is relevant to the event of admission for hospital care, and needs to be reassessed based on events that occur afterwards. For example, a low probability of early delivery may lead to a decision not to administer corticosteroids at admission; however, subsequent ruptured membranes during hospitalization would prompt reassessment of risk. Unfortunately, we did not have detailed follow-up information required to construct a time-varying model for multiple reassessment of the decision about timing of birth. Third, we did not account for inter-centre variability recognising differences between centres, although we tried to limit this by excluding any practice-related predictors to increase generalisability. Fourth, we used internal validation to estimate the generalisability of our model. However, external validation (for example, in a similar cohort of women at risk of preterm delivery, or women admitted at 32–34 weeks for which there were few cases) is needed to better assess the performance of the predictive model in other settings. Finally, we did not have reliable information on pre-pregnancy body mass index and assisted reproductive technology use, which may constitute important risk factors (41.9% and 76.6% of values were missing, respectively).

## Conclusion

In conclusion, we propose a useful tool to improve prediction of delivery within 7 days at the time of admission among women with threatened preterm birth primarily at 24^+0^ and 29^+6^ weeks. Until the discovery of novel biomarkers can improve prediction, such a tool can significantly improve rates of optimal steroid use by increasing the proportion of women who receive steroids within 7 days prior to delivery, and decrease rates of suboptimal steroid use by not immediately treating women with a very low probability of delivery within 7 days

## Additional files

Additional file 1: Table S1. A list of all members of the collaborative group in CPN. (DOCX 13 kb)

Additional file 2: Table S2. Definitions of conditions and variables as used in the Canadian Perinatal Network. (DOCX 13 kb)

Additional file 3:Figure S1. Kaplan-Meier curve showing the proportion of women who remained pregnant from the time that they were admitted to hospital and identified as being at risk of delivery within 7 days. (DOCX 26 kb)

Additional file 4: Table S3. Pregnancy outcomes among women who presented at 24–34 weeks. (DOCX 15 kb)

Additional file 5: Figure. S2. Corrected calibration curve of the final model after internal validation. (DOCX 27 kb)

Additional file 6: Table S4. Sensitivity analysis of the final model after excluding women with missing values. (DOCX 13 kb)

Additional file 7: Table S5. Sensitivity analysis of the final model among singleton and multiple pregnancies. (DOCX 13 kb)

Additional file 8: Table S6. Sensitivity analysis of the final model restricting to women <32 weeks. (DOCX 14 kb)

## Abbreviations

AUC: Area under the curve
CNS: Central nervous system
CPN: Canadian Perinatal Network
GA: Gestational age
LMICs: Low- and middle-income countries
LR-: Negative likeliehood ratio
LR+: Positive likelihood ratio
NPV: Negative predictive value
OR: Odds ratio
PPROM: Preterm pre-labour rupture of membranes
ROC: Receiver operating characteristic [curve]

## References

[1] Public Health Agency of Canada (2008). Canadian Perinatal Health Report.

[2] Magee L, Sawchuck D, Synnes A, von Dadelszen P (2011). Magnesium Sulphate for Fetal Neuroprotection Consensus Committee. SOGC Clinical Practice Guideline. Magnesium sulphate for fetal neuroprotection. J Obstet Gynaecol Can.

[3] Roberts D, Dalziel SR (2006). Antenatal corticosteroids for accelerating fetal lung maturation for women at risk of preterm birth. Cochrane Database Syst Rev.

[4] Kazem M, Hutcheon JA, Joseph KS (2012). A population-based study of antenatal corticosteroid prophylaxis for preterm birth. J Obstet Gynaecol Can.

[5] Crane J, Armson A, Brunner M, De La Ronde S, Farine D, Keenan-Lindsay L (2003). Antenatal corticosteroid therapy for fetal maturation. J Obstet Gynaecol Can.

[6] Gilstrap LC, Christensen R, Clewell WH, D’Alton ME (1995). National Institutes of Health Consensus Development Panel. Effect of corticosteroids for fetal maturation on perinatal outcomes. J Am Med Assoc.

[7] National Institute for Health and Care Excellence. Preterm labour and birth. 2015. Available at: https://www.nice.org.uk/guidance/ng25?unlid=9291036072016213201257. 26632624

[8] Mahony R, McKeating A, Murphy T, McAuliffe F, O’Herlihy C, Foley M (2010). Appropriate antenatal corticosteroid use in women at risk for preterm birth before 34 weeks of gestation. BJOG.

[9] Sanya R, Al Naggar E, Gasim M, Ahmed BI (2014). Use or overuse of antenatal corticosteroids for suspected preterm birth. J Matern Fetal Neonatal Med.

[10] Razaz N, Skoll A, Fahey J, Allen V, Joseph K (2015). Trends in Optimal, Suboptimal, and Questionably Appropriate Receipt of Antenatal Corticosteroid Prophylaxis. Obstet Gynecol.

[11] Crowther CA, McKinlay CJD, Middleton P, Harding JE. Repeat doses of prenatal corticosteroids for women at risk of preterm birth for improving neonatal health outcomes. Cochrane Database Syst Rev. 2015;(7): CD003935. 10.1002/14651858.CD003935.pub4 PMC7104525 26142898

[12] Murphy KE, Hannah ME, Willan AR (2008). Multiple courses of antenatal corticosteroids for preterm birth (MACS): a randomised controlled trial. Lancet.

[13] French NP, Hagan R, Evans SF, Godfrey M, Newnham JP (1999). Repeated antenatal corticosteroids: Size at birth and subsequent development. Am J Obstet Gynecol.

[14] Stalnacke J, Diaz Heijtz R, Norberg H, Norman M, Smedler AC, Forssberg H (2013). Cognitive outcome in adolscents and young adults after repeat courses of antenatal corticosteroids. J Pediatr.

[15] Asztalos EV, Murphy KE, Willan AR (2013). Multiple Courses of Antenatal Corticosteroids for Preterm Birth Study: Outcomes in Children at 5 Years of Age (MACS-5). JAMA Pediatr.

[16] Asztalos E, Willan A, Murphy K (2014). Association between gestational age at birth, antenatal corticosteroids, and outcomes at 5 years: multiple courses of antenatal corticosteroids for preterm birth study at 5 years of age (MACS-5). BMC Pregnancy Childbirth.

[17] Althabe F, Belizán JM, EM MC (2015). A population-based, multifaceted strategy to implement antenatal corticosteroid treatment versus standard care for the reduction of neonatal mortality due to preterm birth in low-income and middle-income countries: the ACT cluster-randomised trial. Lancet.

[18] Honest H, Forbes CA, Durée KH (2009). Screening to prevent spontaneous preterm birth: systematic reviews of accuracy and effectiveness literature with economic modelling. Health Technol Assess.

[19] Honest H, Bachmann LM, Sundaram R, Gupta JK, Kleijnen J, Khan KS (2004). The accuracy of risk scores in predicting preterm birth – a systematic review. J Obstet Gynaecol.

[20] Boots AB, Sanchez-Ramos L, Bowers DM, Kaunitz AM, Zamora J, Schlattmann P (2014). The short-term prediction of preterm birth: a systematic review and diagnostic metaanalysis. Am J Obstet Gynecol.

[21] Esplin MS, Elovitz MA, Iams JD (2017). Predictive Accuracy of Serial Transvaginal Cervical Lengths and Quantitative Vaginal Fetal Fibronectin Levels for Spontaneous Preterm Birth Among Nulliparous Women. JAMA.

[22] Conde-Agudelo A, Papageorghiou A, Kennedy S, Villar J (2011). Novel biomarkers for the prediction of the spontaneous preterm birth phenotype: a systematic review and meta-analysis. BJOG.

[23] Berghella V, Saccone G (2016). Fetal fibronectin testing for prevention of preterm birth in singleton pregnancies with threatened preterm labor: a systematic review and metaanalysis of randomized controlled trials. Am J Obstet Gynecol.

[24] Macones GA (2016). Fetal fibronectin testing in threatened preterm labor: time to stop. Am J Obstet Gynecol.

[25] Magee LA, von Dadelszen P, Allen VM (2011). The Canadian Perinatal Network: A National Network Focused on Threatened Preterm Birth at 22 to 28 Weeks’ Gestation. J Obstet Gynaecol Can.

[26] Allouche M, Huissoud C, Guyard-Boileau B, Rouzier R, Parant O (2011). Development and validation of nomograms for predicting preterm delivery. Am J Obstet Gynecol.

[27] Moons KGM, Altman DG, Vergouwe Y, Royston P (2009). Prognosis and prognostic research: application and impact of prognostic models in clinical practice. BMJ.

[28] Moons KGM, Royston P, Vergouwe Y, Grobbee DE, Altman DG (2009). Prognosis and prognostic research: what, why, and how?. BMJ.

[29] Steyerberg EW (2009). Clinical Prediction Models: A Practical Approach to Development, Validation, and Updating.

[30] Nykjaer C, Alwan NA, Greenwood DC, Simpson NAB, Hay AWM, White KLM, Cade JE (2014). Maternal alcohol intake prior to and during pregnancy and risk of adverse birth outcomes: evidence from a British cohort. J Epidemiol Community Health.

[31] Bhattacharya S, Lowit A, Bhattacharya S, Raja EA, Lee AJ, Mahmood T, Templeton A (2012). Reproductive outcomes following induced abortion: a national register-based cohort study in Scotland. BMJ Open.

[32] Goldenberg RL, Culhane JF, Iams JD, Romero R (2008). Epidemiology and causes of preterm birth. Lancet.

[33] Vergouwe Y, Royston P, Moons KGM, Altman DG (2010). Development and validation of a prediction model with missing predictor data: a practical approach. J Clin Epidemiol.

[34] Royston P, Moons KGM, Altman DG, Vergouwe Y (2009). Prognosis and prognostic research: Developing a prognostic model. BMJ.

[35] Malone FD, Canick JA, Ball RH (2005). First-Trimester or Second-Trimester Screening, or Both, for Down’s Syndrome. N Engl J Med.

[36] Wapner R, Thom E, Simpson JL (2003). First-Trimester Screening for Trisomies 21 and 18. N Engl J Med.

[37] Wald NJ, Rodeck C, Hackshaw AK, Walters J, Chitty L, Mackinson AM, SURUSS Research Group. First and second trimester antenatal screening for Down’s syndrome: the results of the Serum, Urine and Ultrasound Screening Study (SURUSS). J Med Screen 2003; 10(4): 56–104. 10.1258/096914103321824133 14746340

[38] Spencer K, Spencer CE, Power M, Dawson C, Nicolaides KH (2003). Screening for chromosomal abnormalities in the first trimester using ultrasound and maternal serum biochemistry in a one-stop clinic: a review of three years prospective experience. BJOG.

[39] van de Mheen L, Schuit E, Lim AC (2014). Prediction of preterm birth in multiple pregnancies: development of a multivariable model including cervical length measurement at 16 to 21 weeks’ gestation. J Obstet Gynaecol Can.

