# Supplementary material for: Timing of delivery in a high-risk obstetric population: a clinical prediction model
Source: BMC Pregnancy Childbirth. 2017 Jun 29;17:202. doi: 10.1186/s12884-017-1390-9 (PMC5492352; doi:10.1186/s12884-017-1390-9)
Supplement: Supplementary file 1 — A list of all members of the collaborative group in CPN. (DOCX 13 kb) [file 12884_2017_1390_MOESM1_ESM.docx]

| **CPN Collaborative Group** |
| --- |
| ***Steering Committee****:* Laura A. Magee (St. George’s University of London, London UK), Robert Liston (University of British Columbia, Vancouver BC), Victoria Allen (Dalhousie University, Halifax NS), Mark Ansermino (University of British Columbia, Vancouver BC), François Audibert (Université de Montréal, Montreal QC), Rollin Brant (University of British Columbia, Vancouver BC), Emmanuel Bujold (Université Laval, Québec QC), Joan Crane (Memorial University of Newfoundland, St. John's NF), Nestor Demianczuk (University of Alberta, Edmonton AB), KS Joseph (University of British Columbia, Vancouver BC), Jean-Marie Moutquin (Université de Sherbrooke, Sherbrooke QC), Bruno Piedboeuf (Université Laval, Québec QC), Graeme Smith (Queen’s University at Kingston, Kingston ON), Peter von Dadelszen (St. George’s University of London, London UK), Mark Walker (University of Ottawa, Ottawa ON), Wendy Whittle (University of Toronto, Toronto ON) |
| ***Data Team & Co-ordinator****:* Larry Li (programmer), Tang Lee (analyst), Dane A. De Silva (co-ordinator) |
| *Site Investigators & Co-ordinators:* Laura A. Magee and Peter von Dadelszen (BC Women’s Hospital & Health Centre, Vancouver BC), Jerome Dansereau (Victoria General Hospital, Victoria BC), Nestor Demianczuk & Cheryl Lux-Warholik (Royal Alexandra Hospital, Edmonton AB), Stephen Wood & Lorel Dederer (Foothills Medical Centre, Calgary AB), Femi Olatunbosun & Terri Shewchuk (Royal University Hospital, Saskatoon SK), George Carson, (Regina General Hospital, Regina SK), Graeme Smith & Heather Ramshaw (Kingston General Hospital, Kingston ON), Renato Natale & Laura McMurphy (London Health Sciences Centre, London ON), Wendy Whittle & Karen Muller (Mount Sinai Hospital, Toronto ON), Mark Walker & Ruth White (The Ottawa Hospital, Ottawa ON), Sarah McDonald (McMaster University Medical Centre), François Audibert & Jocelyne Vallée (Centre Hôspitalier Universitaire Sainte-Justine, Montréal QC), Jean-Charles Pasquier (Centre Hôspitalier Universitaire de Sherbrooke, Sherbrooke QC), Emmanuel Bujold & Amélie Tétu (Centre Hôspitalier de L’Université Laval, Québec City QC), Victoria M. Allen & Venessa Ryan (IWK Health Centre, Halifax NS), Joan M.G. Crane & Donna Hutchens (Women's Health Program, Eastern Health, St. John’s NL) |

**Table S1:** Members of the CPN (Canadian Perinatal Network) Collaborative Group
