# Supplementary material for: Timing of delivery in a high-risk obstetric population: a clinical prediction model
Source: BMC Pregnancy Childbirth. 2017 Jun 29;17:202. doi: 10.1186/s12884-017-1390-9 (PMC5492352; doi:10.1186/s12884-017-1390-9)
Supplement: Supplementary file 2 — Definitions of conditions and variables as used in the Canadian Perinatal Network. (DOCX 13 kb) [file 12884_2017_1390_MOESM2_ESM.docx]

**Table S2.** Definitions of conditions and variables as used in the Canadian Perinatal Network.

| **Condition / Variable** | **Definition** |
| --- | --- |
| Preterm labour | Regular contractions every 5 minutes or more frequently, with documented cervical change at <37^+0^ weeks gestation. |
| Preterm pre-labour rupture of membranes (PPROM) | Pre-labour rupture of membranes confirmed by positive ferning and/or pooling of amniotic fluid prior to onset of regular contractions at <37^+0^ weeks gestation. |
| Short cervix without uterine contractions | Cervical shortening (≤10 mm by vaginal ultrasonography) without regular contractions (every 5 minutes or more frequently) |
| Dilated cervix or prolapsed membranes without uterine contractions | Prolapsed membranes at or beyond the external os as visualized on speculum exam, or any cervical dilatation of external os by endovaginal ultrasonography, without regular contractions. |
| Antepartum haemorrhage | >15 mL of vaginal bleeding prior to the onset of labour. |
| Smoking during pregnancy | Any smoking during this pregnancy |
