# Supplementary material for: Timing of delivery in a high-risk obstetric population: a clinical prediction model
Source: BMC Pregnancy Childbirth. 2017 Jun 29;17:202. doi: 10.1186/s12884-017-1390-9 (PMC5492352; doi:10.1186/s12884-017-1390-9)
Supplement: Supplementary file 3 — Kaplan-Meier curve showing the proportion of women who remained pregnant from the time that they were admitted to hospital and identified as being at risk of delivery within 7 days. (DOCX 26 kb) [file 12884_2017_1390_MOESM3_ESM.docx]

**Figure S1:** Kaplan-Meier curve showing the proportion of women who remained pregnant from the time that they were admitted to hospital and identified as being at risk of delivery within 7 days
