# Supplementary material for: Timing of delivery in a high-risk obstetric population: a clinical prediction model
Source: BMC Pregnancy Childbirth. 2017 Jun 29;17:202. doi: 10.1186/s12884-017-1390-9 (PMC5492352; doi:10.1186/s12884-017-1390-9)
Supplement: Supplementary file 4 — Pregnancy outcomes among women who presented at 24–34 weeks. (DOCX 15 kb) [file 12884_2017_1390_MOESM4_ESM.docx]

**Table S3:** Pregnancy outcomes among women who presented at 24-34 weeks with threatened preterm birth (N (%) women or median [25th, 75th centiles])

|  | **Delivery within 7 days (N=1473)** | **Delivery at >7days**  **(N=1539)** | **p value** |
| --- | --- | --- | --- |
| **Maternal interventions prior to delivery OR ON ADMISSION** | | | |
| Maternal transport prior to delivery | 861 (58.5%) | 523 (34.0%) | **<0.001** |
| Antibiotics | 995 (67.5%) | 573 (37.2%) | **<0.001** |
| Antenatal corticosteroids on admission | 846 (57.4%) | 941 (61.1%) | **<0.001** |
| Tocolysis | 467 (31.7%) | 238 (15.5%) | **<0.001** |
| Cervical cerclage in place | 124 (8.4%) | 128 (8.3%) | 0.973 |
| Fetal fibronectin testing with a known result | 67 (4.5%) | 115 (7.5%) | **<0.001** |
| **PREGNANCY OUTCOMES** |  |  |  |
| Number of hospital admissions per woman | 1 [1,1] | 2 [1,2] | **<0.001** |
| Length of stay (days) | 4 [3, 6] | 13 [3,25] | **<0.001** |
| Preterm birth < 37 weeks | 1473 (100.0%) | 1110 (72.1%) | **<0.001** |
| Preterm birth <29 weeks | 1350 (91.6%) | 324 (21.1%) | **<0.001** |
| GA at delivery (weeks) | 27 [25, 28] | 33 [29, 37] | **<0.001** |
| Type of delivery |  |  |  |
| Spontaneous birth |  |  |  |
| Iatrogenic birth |  |  |  |
| Induction of labour | 37 (2.5%) | 226 (14.7%) | **<0.001** |
| Caesarean section | 663 (45.0%) | 663 (43.1%) | 0.303 |
| Latency - admission to delivery (days) | 1 [0,4] | 44 [19, 73.5] | **<0.001** |
| 0-2 | 936 (63.5%) | 0 |  |
| 3 – 7 | 537 (36.5%) | 0 |  |
| 8-14 | 0 | 265 (17.2%) |  |
| 15-21 | 0 | 174 (11.3%) |  |
| 22-28 | 0 | 113 (7.3%) |  |
| >28 | 0 | 987 (64.1%) |  |
| Severe Maternal Morbidity (one or more)‡ | 41 (2.8%) | 15 (1.0%) | **<0.001** |
| Cardiovascular | 0 | 1 (0.1%) | 0.999 |
| Respiratory | 20 (1.4%) | 6 (0.4%) | **0.008** |
| CNS | 0 | 0 | 0.999 |
| Renal | 1 (0.1%) | 0 | 0.489 |
| Hematological | 3 (0.2%) | 1 (0.1%) | 0.364 |
| Hepatic | 5 (0.3%) | 1 (0.1%) | 0.117 |
| Infection | 19 (1.3%) | 8 (0.5%) | **0.041** |
| **NEONATAL OUTCOMES** | N=1749 infants | N=1954 infants |  |
| Perinatal mortality | 119 (6.8%) | 15 (0.8%) | **<0.001** |
| Stillbirth | 52 (3.0%) | 9 (0.5%) | **<0.001** |
| Neonatal death | 68 (3.9%) | 6 (0.3%) | **<0.001** |
| Birth weight <10^th^ centile | 61 (3.5%) | 167 (8.5%) | **<0.001** |
| Birth weight <3^rd^ centile | 21 (1.2%) | 50 (2.6%) | **0.004** |
| *Missing* | 495 (28.3%) | 372 (19.0%) |  |
| Admission to NICU | 1633 (93.4%) | 1251 (64.0%) | **<0.001** |
| Length of stay at NICU (days) | 57 [24, 86] | 23 [9, 52] | **<0.001** |
| *Missing* | 13 (0.7%) | 15 (0.8%) |  |

‡ Severe maternal morbidity includes one or more of cardiovascular events (including use of third injectable antihypertensive, positive inotropic support, or myocardial ischemia/infarction), respiratory events (including use of intubation, non-invasive ventilation, pulmonary edema, or requirement of >50% oxygen), CNS events (including blindness, eclampsia, GCS<13, stroke), renal events (including acute renal failure, dialysis), haematological events (including disseminated intravascular coagulation, hysterectomy, uterine artery embolization), hepatic events (including hepatic failure, hepatic rupture, thromboembolism), or infection (including sepsis of endometritis).
