# Supplementary material for: Timing of delivery in a high-risk obstetric population: a clinical prediction model
Source: BMC Pregnancy Childbirth. 2017 Jun 29;17:202. doi: 10.1186/s12884-017-1390-9 (PMC5492352; doi:10.1186/s12884-017-1390-9)
Supplement: Supplementary file 5 — Corrected calibration curve of the final model after internal validation. (DOCX 27 kb) [file 12884_2017_1390_MOESM5_ESM.docx]

**Figure S2:** Corrected calibration curve of the final model after internal validation
