# Supplementary material for: Timing of delivery in a high-risk obstetric population: a clinical prediction model
Source: BMC Pregnancy Childbirth. 2017 Jun 29;17:202. doi: 10.1186/s12884-017-1390-9 (PMC5492352; doi:10.1186/s12884-017-1390-9)
Supplement: Supplementary file 6 — Sensitivity analysis of the final model after excluding women with missing values. (DOCX 13 kb) [file 12884_2017_1390_MOESM6_ESM.docx]

**Table S4.** Sensitivity analyses of the final model predicting delivery within 7 days after admission excluding 23 women with missing values.

| **Risk factor** | **OR [95% CI]** | **Adjusted OR* (95% CI)** |
| --- | --- | --- |
| Maternal age (years) |  |  |
| <40 | Reference | Reference |
| ≥40 | 0.55 [0.39, 0.78] | 0.66 [0.45, 0.96] |
| Parity |  |  |
| Nulliparous | Reference | Reference |
| Parity ≥1 | 0.68 [0.59, 0.78] | 0.57 [0.49, 0.67] |
| Smoking during pregnancy† | 1.36 [1.13, 1.63] | 1.38 [1.13, 1.69] |
| Gestational age (GA) on admission‡ | 1.08 [1.03, 1.13] | ‡ |
| Maternal conditions |  |  |
| Preterm labour | 2.38 [2.05, 2.76] | 7.37 [5.84, 9.30] |
| PPROM | 1.52 [1.30, 1.76] | 5.68 [4.49, 7.19] |
| Prolapsed membranes | 1.75 [1.41, 2.18] | 6.33 [4.74, 8.45] |
| Associated antepartum hemorrhage | 2.06 [1.63, 2.60] | 2.01 [1.56, 2.59] |

OR denotes odds ratio, PPROM denotes preterm pre-labour rupture of membranes

* adjusted for all other factors presented in the table

Equation:

‡ gestational age was modelled using higher order polynomials (see the equation below)

† 23 missing values

Risk Score=12.98 – [0.42 × Maternal age] – [1.14 × GA] + [0.02 × (GA)^2^] – [0.55 × Parity] + [0.32 × Smoking] + [2.00 × Preterm labour] + [1.74 × PPROM] + [1.85 × Prolapsed membranes] + [0.70 × Antepartum haemorrhage]
