# Supplementary material for: Timing of delivery in a high-risk obstetric population: a clinical prediction model
Source: BMC Pregnancy Childbirth. 2017 Jun 29;17:202. doi: 10.1186/s12884-017-1390-9 (PMC5492352; doi:10.1186/s12884-017-1390-9)
Supplement: Supplementary file 7 — Sensitivity analysis of the final model among singleton and multiple pregnancies. (DOCX 13 kb) [file 12884_2017_1390_MOESM7_ESM.docx]

**Table S5.** Sensitivity analyses of the final model predicting delivery within 7 days after admission among singleton and multiple pregnancies.

| **Risk factor** | **Adjusted OR [95% CI]** Singletons (N=2356) | **Adjusted OR [95% CI]** Multiples (N=633) |
| --- | --- | --- |
| Maternal age (yr) |  |  |
| <40 | Reference | Reference |
| ≥40 | 0.70 [0.45 – 1.07] | 0.63 [0.26 – 1.53] |
| Parity |  |  |
| Nulliparous | Reference | Reference |
| Parity ≥1 | 0.57 [0.48 – 0.68] | 0.54 [0.37 – 0.81] |
| Smoking during pregnancy* | 1.31 [1.06 – 1.62] | 1.68 [0.97 – 2.91] |
| Gestational age (GA) on admission | ‡ | ‡ |
| Maternal conditions |  |  |
| Preterm labour | 6.72 [5.16 – 8.76] | 8.24 [5.00 – 13.56] |
| PPROM | 4.63 [3.55 – 6.05] | 10.79 [6.33 – 18.40] |
| Prolapsed membranes | 6.03 [4.36 – 8.34] | 6.00 [3.12 – 11.53] |
| Associated antepartum haemorrhage | 1.95 [1.49 – 2.55] | 2.04 [0.94 – 4.46] |
| **AUC** | 0.71 [0.69 – 0.73] | 0.77 [0.73 – 0.81] |

*Smoking not imputed

‡ gestational age was modelled using higher order polynomials
