# Supplementary material for: Timing of delivery in a high-risk obstetric population: a clinical prediction model
Source: BMC Pregnancy Childbirth. 2017 Jun 29;17:202. doi: 10.1186/s12884-017-1390-9 (PMC5492352; doi:10.1186/s12884-017-1390-9)
Supplement: Supplementary file 8 — Sensitivity analysis of the final model restricting to women <32 weeks. (DOCX 14 kb) [file 12884_2017_1390_MOESM8_ESM.docx]

**Table S6.** Sensitivity analyses of the final model predicting delivery within 7 days after admission among women at <32 weeks.

| **Risk factor** | **Adjusted OR [95% CI]** GA <32 wks (N=2965) |
| --- | --- |
| Maternal age (yr) |  |
| <40 | Reference |
| ≥40 | 0.65 [0.45 – 0.96] |
| Parity |  |
| Nulliparous | Reference |
| Parity ≥1 | 0.57 [0.49 – 0.67] |
| Smoking during pregnancy* | 1.38 [1.13 – 1.68] |
| Gestational age (GA) on admission | ‡ |
| Maternal conditions |  |
| Preterm labour | 7.40 [5.87 – 9.35] |
| PPROM | 5.64 [4.46 – 7.15] |
| Prolapsed membranes | 6.31 [4.73 – 8.43] |
| Associated antepartum haemorrhage | 2.01 [1.56 – 2.59] |
| **AUC** | 0.72 [0.71 – 0.74] |

*Smoking not imputed

‡ gestational age was modelled using higher order polynomials
